# Clinical outcomes and radiologic parameters of percutaneous endoscopic posterior/transforaminal lumbar interbody fusion using rammed-earth technique

**DOI:** 10.3389/fsurg.2026.1822943

**Published:** 2026-05-15

**Authors:** Qingyang Kang, Zhilin Ge, Jiheng Zhan, Xuzhou Li, Guanwei Huang, Guoyi Su

**Affiliations:** 1 The Second Clinical College of Guangzhou University of Chinese Medicine, Guangzhou, Guangdong, China; 2 Department of Spinal Surgery, Guangdong Provincial Hospital of Chinese Medicine, Guangzhou, Guangdong, China

**Keywords:** endoscopic surgery, lumbar, minimally invasive surgical procedures, spine, spondylosis

## Abstract

**Introduction:**

To assess the clinical effectiveness of employing rammed-earth technique in bone grafting during percutaneous endoscopic posterior/transforaminal lumbar interbody fusion (PE-P/TLIF) in treating lumbar degenerative diseases.

**Methods:**

This retrospective study analyzed patients who underwent PE-P/TLIF for lumbar degenerative diseases from December 2019 to August 2023. The study included 22 patients treated with the rammed-earth technique and 21 patients treated without the rammed-earth technique. Surgical time, intraoperative blood loss, and the number of surgical segments were compared between the two groups. Pain and neurological function were evaluated using the Visual Analog Scale (VAS), the Oswestry Disability Index (ODI), and the Japanese Orthopaedic Association (JOA) lumbar score. Radiographic measurements, including lumbar lordosis, segmental lordosis, intervertebral height, and cage subsidence depth.

**Results:**

Both groups showed varying degrees of reduction in VAS and ODI scores from preoperative levels on the first postoperative day and at the final follow-up, while the JOA scores increased significantly. At the final follow-up, the rammed group had higher JOA ratings than the umrammed group. (*P* < 0.05). Regarding radiographic parameters, the postoperative intervertebral height was greater than preoperative and at the final follow-up (*P* < 0.05). The rammed group showed significantly lower rates and depth of cage subsidence compared to the unrammed group (*P* < 0.05).

**Conclusion:**

Using the rammed-earth technique in PE-P/TLIF procedures can securely reduce the rate and depth of cage subsidence, improving lumbar spine function after a follow-up period of at least six months.

## 1 Introduction

Lumbar degenerative disease (LDD) is highly prevalent in the senile population and is often accompanied by functional impairments such as claudication and decreased lumbar mobility (1, 2). Percutaneous endoscopic posterior/transforaminal lumbar interbody fusion (PE-P/TLIF) has emerged as a promising technique for treating LDD, offering advantages over traditional open posterior lumbar interbody fusion (PLIF) including minimized tissue disruption, reduced intraoperative blood loss, shorter hospitalizations, decreased surgical complications and reliable fusion rate (3, 4).

Despite these benefits, several studies have reported varying degrees of interbody cage subsidence into the endplates during postoperative follow-up of endoscopic lumbar fusion procedures, potentially leading to recurrent pain, neurological deficits, and other adverse outcomes (5, 6). According to previous reports, the incidence of cage subsidence in endoscopic lumbar fusion surgery can be up to 79.5% (7). Currently, due to the relatively new adoption of PE-P/TLIF, there is still a lack of studies evaluating the incidence of cage subsidence (8). The prevention of cage subsidence remains a critical factor in improving surgical outcomes. Previous strategies have primarily focused on the use of 3D-printed cages or larger interbody cages. However, 3D-printed cages are costly, and the use of larger cages during endoscopic fusion procedures may increase the risk of nerve root injury. Moreover, oversized cages may raise the risk of spinal canal invasion (4, 9–11).

Therefore, we employed the rammed-earth technique. It can enhance anterior column mechanical support, compensating for the smaller cage size commonly used in endoscopic fusion. Meanwhile, this approach offers substantial practical advantages, including easy operation, cost—effectiveness, and the avoidance of nerve injury and spinal canal compromise risks that are associated with the implantation of larger cages. This retrospective study analyzes the outcomes of utilizing the rammed-earth technique during PE-P/TLIF in LDD management.

## 2 Materials and methods

### 2.1 Patients

This study analyzed patients who underwent PE-P/TLIF for lumbar degenerative disease between December 2019 and August 2023. The inclusion criteria included: (1) patients with a confirmed diagnosis of LDD and exhibiting clear surgical indications; (2) absence of severe lumbar trauma or a history of lumbar operation. The exclusion criteria were: (1) previous spinal surgery or concurrent spinal disorders including infection, tumor, extreme deformities; (2) other conditions that could cause low back pain or lower limb dysfunction, such as intermittent claudication due to cerebrovascular accidents, lower extremity arthritis, lower extremity surgery; (3) poor general health, particularly severe cardiopulmonary issues; (4) diagnosed as mental nervous system diseases, including dementia, aphasia, etc.; (5) inability to adhere to a minimum follow-up period of six months. This study was approved by the Ethics Committee of Guangdong Provincial Hospital of Chinese Medicine (ZE2024-338-01).

Patients who underwent the PE-P/TLIF procedure with the use of the rammed-earth technique were classified as the rammed group (*n* = 22), while those who did not receive this technique were classified as the unrammed group (*n* = 21). Demographic and clinical baseline information was gathered for all patients, including gender, age, height, weight, BMI, length of hospital stay, postoperative discharge time, surgery duration, intraoperative blood loss, and quantity of fusion levels.

### 2.2 Surgical procedures

Prior to surgery, the patient was positioned in the prone position and administered general anesthesia. C-arm fluoroscopy was used to identify the precise surface projections of the pedicles at both cranial and caudal ends of the fusion segment, guiding incision placement and surgical approach. Under fluoroscopic guidance, bilateral pedicle guidewires were inserted. Percutaneous pedicle screws were initially placed on the non-decompression side to provide structural stability, while the guidewire on the decompression side was retained for further manipulation.

Subsequently, an incision was made laterally to the superficial projection of the caudal pedicle on the affected side. Dilators were gradually introduced through the soft tissues to the surgical site. The inferior articular process of the cranial vertebra was identified to ensure the beveled opening of the working sheath was securely placed against the medial rim. Once positioned, a trephine was introduced to make minor bone cuts on the inferior articular process, which were confirmed under fluoroscopy.

Under endoscopic visualization, surrounding soft tissues were dissected, and the articular process and partial lamina were resected as per decompression and fusion cage requirements. The ipsilateral nerve root canal and lateral recess were expanded. The annulus fibrosus of the intervertebral disc was incised to remove the nucleus pulposus. During preparation of the cartilaginous endplates, an endoscopic chisel or curette was used to abrade the surface until slight bone bleeding was observed. After withdrawing the endoscope, intervertebral bone grafting was performed using a combination of autograft and allograft bone.

**Figure 1:**
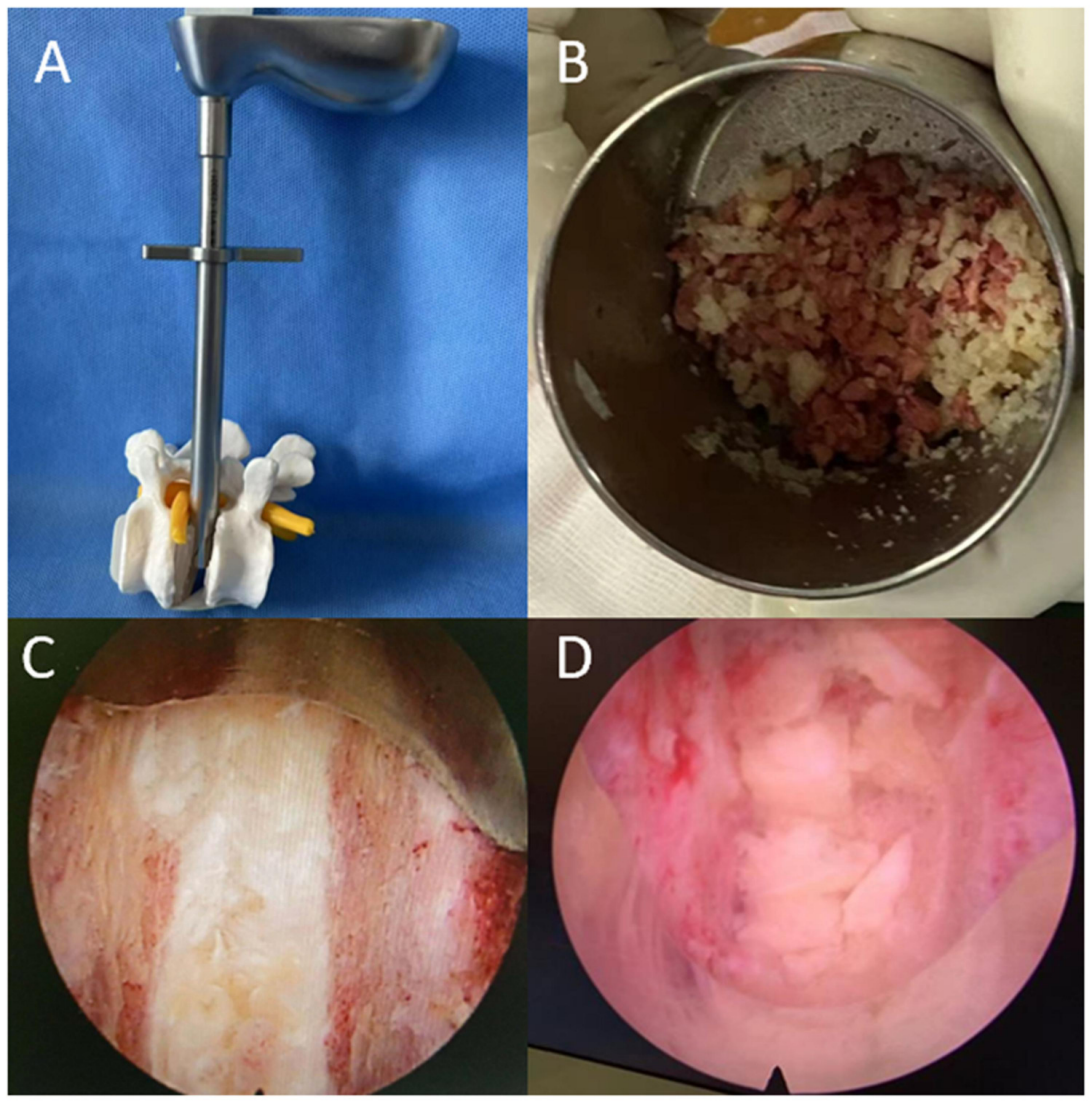
The use of rammed-earth technique. **(A)** Bone graft funnel inserted into the working channel. **(B)** Mixture of autograft and allograft. **(C)** Endoscopic view showing the anterior vertebral line and anterior longitudinal ligament. **(D)** Endoscopic appearance of bone graft compaction following the application of the rammed-earth technique.

In the rammed group, a working cannula was first inserted into the intervertebral space to achieve initial distraction, typically expanding the disc height to approximately 11–12 mm. Intervertebral space preparation was performed to cover nearly two-thirds of the disc area. A total of 8–10 g of pre-mixed bone graft (autograft and allograft) was divided into three portions (each 3 g). The 8–10 g of pre-mixed bone graft represents an optimal average target rather than a rigid absolute mass. The precise implanted volume was titrated intraoperatively based on each patient's unique anatomical dimensions, including native disc height and endplate footprint. The first portion was delivered into the disc space using a calibrated funnel, filling one-third of the space. The remaining two portions were inserted with the aid of a tamper rod placed within the funnel, and each was gently impacted with approximately 3–4 light taps to compact the graft anteriorly. Care was taken to monitor the depth markings to avoid breaching the anterior annulus fibrosus. Endoscopic visualization confirmed adequate graft compaction (Figure 1). If the graft remained loose, the tapping process was repeated until satisfactory packing was achieved. The unrammed group followed conventional grafting methods. An appropriately sized cage (22–26 mm × 9 mm × 8–14 mm) was inserted and verified using fluoroscopy.

The endoscope was then reintroduced to remove the dorsal ligamentum flavum and decompress the contralateral side, ensuring thorough decompression of the nerve roots. After hemostasis with radiofrequency, the working sheath was withdrawn.

Finally, the surgeon inserted percutaneous pedicle screws along the pre-positioned guidewires and secured them with connecting rods under compression. Upon completion, a drainage tube was placed at the decompression site to manage postoperative hematoma and serous fluid, which was removed once the drainage volume fell below 50 mL. Perioperative antibiotic prophylaxis was administered for 24 h to prevent infection. On the first day after operation, patients were encouraged to ambulate while wearing a waist supporter.

### 2.3 Clinical outcome assessment

Trained research staff assessed clinical symptoms, and patients were kept unaware of the study's goals at all times. The Pain Visual Analog Scale (VAS), Oswestry Disability Index (ODI), and Japanese Orthopaedic Association (JOA) scores were utilized to evaluate clinical outcomes at three-time points: preoperatively, postoperatively, and during the last follow-up (minimum six months after surgery).

### 2.4 Clinical symptom assessment

Clinical symptom evaluations were conducted by trained research personnel, with patients remaining unaware of the study's specifics. Clinical symptoms were evaluated at three-time points—preoperatively, postoperatively, and at the last follow-up (minimum six months following surgery) using the VAS, ODI, and JOA scores.

### 2.5 Radiographic parameters

This study analyzed preoperative, postoperative, and final follow-up (minimum of 6 months) DR and CT imaging results. Radiographic evaluations were performed independently by two blinded, experienced orthopedic spine surgeons, and the average of their measurements (with an inter-observer error of less than 10%) was used for subsequent analysis. The measured parameters included the following: Lumbar Lordosis Angle: The angle between the line connecting the superior endplate of L1 and the line connecting the inferior endplate of L5; Segmental Lordosis Angle: The angle between the line connecting the superior endplate of the fused segment and the line connecting the inferior endplate of the fused segment; Intervertebral Height: The part of the center line of the superior and inferior adjacent vertebrae outside the vertebral body (12); Fusion-edge: The shortest distance between the posterior aspect of the fusion cage and the posterior edge of the lower adjacent vertebra; Subsidence Depth: The distance from the lowest point of the fusion cage's inferior edge to the superior endplate of the vertebra. Subsidence was defined as more than 2 mm of graft material sinking beneath the endplates.

### 2.6 Sample size and statistical analysis

To determine the required sample size, an *a priori* power analysis was performed using G*Power 3.1 software with an “*F*-test, ANOVA: Repeated measures, within-between interaction” design. The significance level (α) was set at 0.05, and the statistical power was set at 0.9. Based on the first five patients in each group, the calculated within-group correlation coefficient was 0.48, and the effect size was set at 0.25. The analysis indicated that a minimum of 19 subjects per group would be required.

Statistical analyses were performed using SPSS 26.0 (IBM Corp, Armonk, NY), with significance set at *P* < 0.05. Continuous variables were strictly assessed for normality of distribution utilizing the Shapiro–Wilk test prior to further comparative analysis. Variables conforming to a normal distribution are expressed as means with standard deviations (Mean ± SD) and were analyzed using the independent samples *t*-test for continuous between-group comparisons. Conversely, variables demonstrating a non-normal, skewed distribution are presented as medians accompanied by interquartile ranges (IQR) and were subsequently evaluated utilizing the non-parametric Mann–Whitney *U*-test. Categorical variables compared using the Pearson Chi-square test. A 2 × 3 (group × time) repeated measures analysis was conducted to assess the main effects and interactions of the group (two study groups) and time (pre-surgery, post-surgery, last follow-up) factors for each outcome variable. The Greenhouse-Geisser correction was applied when the assumption of sphericity was violated.

## 3 Results

**Figure 2:**
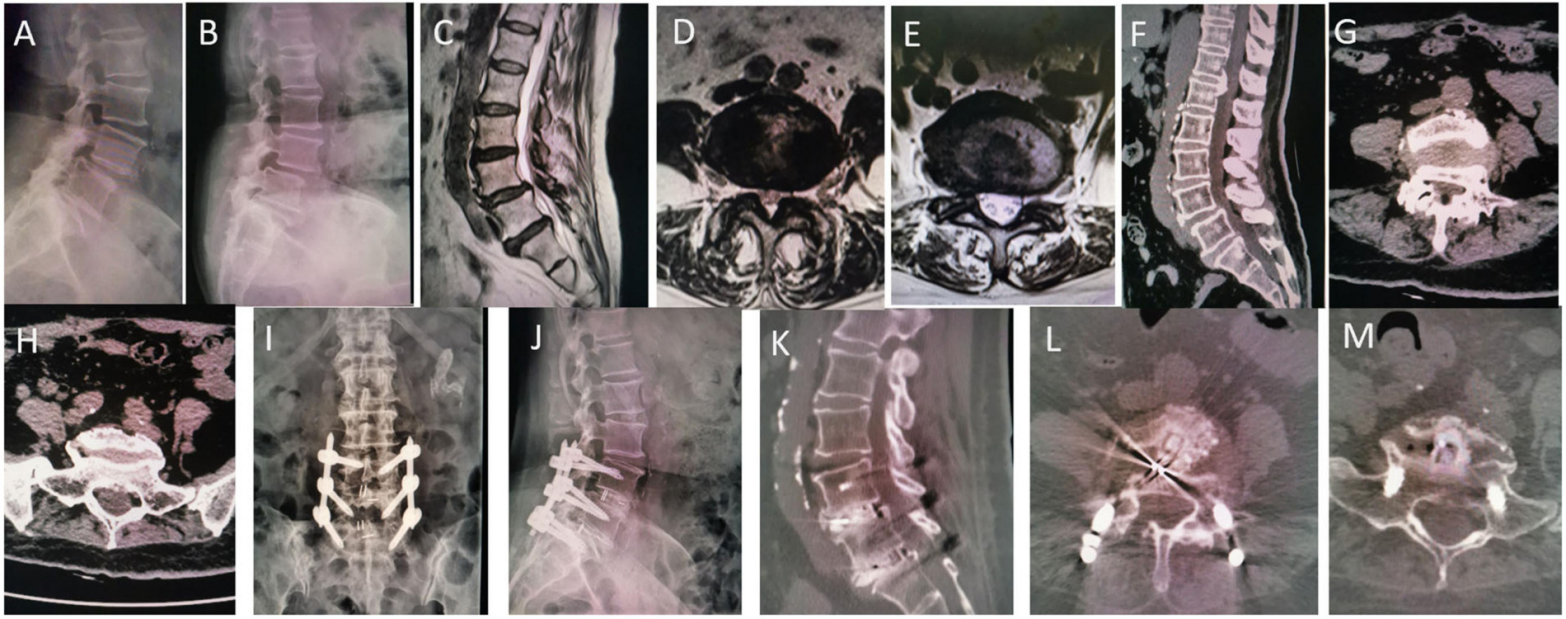
A typical case. A 69-year-old woman presented with low back pain and radiation-induced pain in both lower limbs for 5 years, aggravated for 2 months. **(A–H)** L4 vertebra spondylolisthesis, L4/5 and L5/S1 disc herniation with ligamentum flavum hypertrophic, and spinal canal stenosis. **(C–E)** Postoperative images using rammed-earth technique. **(I–M)** Follow-up after six months. **(K–M)** Extensive bone graft and solid fusion without cage subsidence or endplate collapse.

There were 43 individuals in all who had surgery included in this retrospection study, with 22 patients in the group utilizing the rammed-earth technique and 21 in the group that did not. Based on the surgical segments, 38 patients underwent a single-segment procedure, while 5 patients underwent a two-segment procedure. A typical case is presented in Figure 2. Table 1 outlines the patients' demographic and baseline characteristics. There were no statistically significant differences in the preoperative demographic data between the two groups (*P* > 0.05). All surgical procedures were completed with no complications such as neurological deficits or fixation device failures observed during the perioperative period or follow-up. There were no statistically significant differences between the two groups in terms of hospital stay duration, postoperative discharge time, operative time, intraoperative blood loss, or the quantity of fusion levels (*P* > 0.05).

**Table 1:** Comparative analysis of demographics.

Baseline	Unrammed	Rammed	*P*-value
Age (yr)	57.3 ± 10.8	59.2 ± 11.3	0.968
Weight (kg)	66.2 ± 11.2	63.4 ± 11.6	0.613
Height (cm)	160 (157–164)	161 (154–168)	0.903
BMI (kg/m^2^)	25.6 (22.7–28.0)	24.9 (22.4–26.5)	0.350
Operative Time (min)	255.2 ± 74.0	221 ± 61.2	0.148
Hospital Duration (d)	10 (8.5–11)	10 (8–12)	0.704
Discharge Time (d)	5 (4–6)	5 (4–6)	0.637
Blood Loss (mL)	60 (45–100)	70 (50–100)	0.940
Sex Male/Female	5/16	8/14	0.370
Fusion Levels 1/2	20/1	18/4	0.803

Values are presented as mean ± SD or median (interquartile range).

BMI, body mass index.

### 3.1 Clinical outcome results

**Figure 3:**
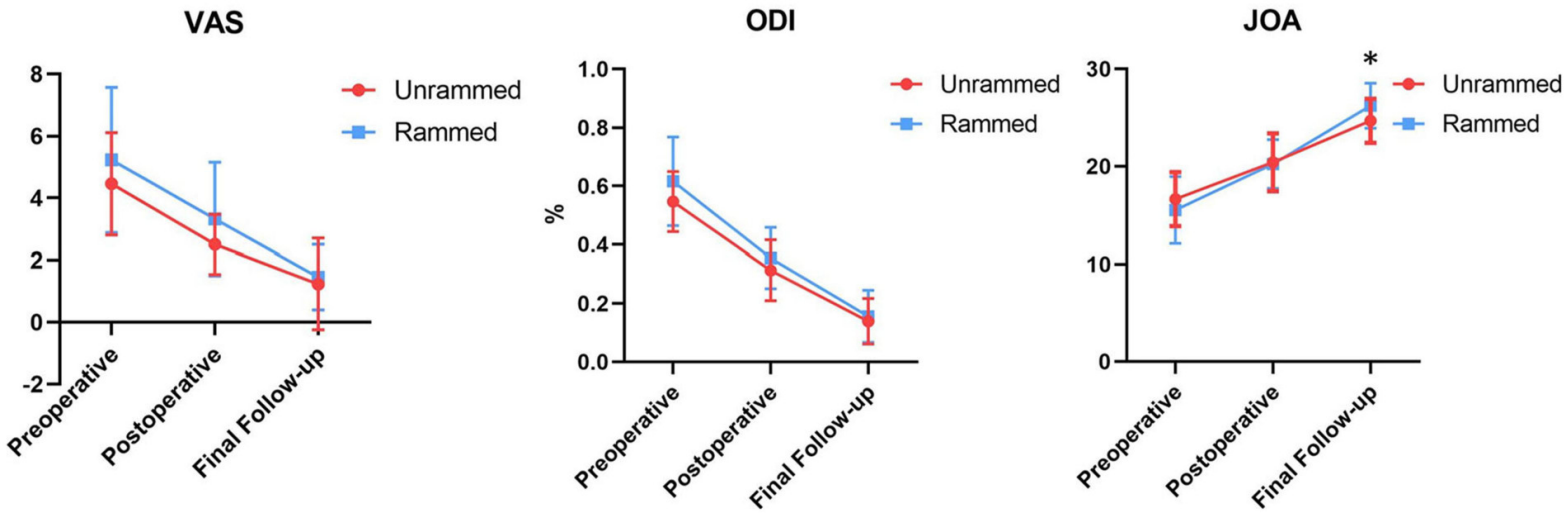
Clinical outcomes charts. In the final follow-up, JOA score showed differences between groups. *P < 0.05.

Both groups showed reduction in VAS and ODI scores at both the first postoperative day and the final follow-up (minimum of six months) compared to preoperative scores (*P* < 0.05). However, there were no statistically significant differences between the two groups in VAS and ODI scores at the same time points (*P* > 0.05). Preoperative and first-day postoperative JOA scores did not significantly differ between the rammed and unrammed groups (*P* > 0.05), but a significant difference was observed at the final follow-up (*P* < 0.05) (Figure 3, Table 2).

**Table 2:** Indicators related to efficacy evaluation.

Scale	Unrammed	Rammed	*P*-value
VAS			
Preoperative	4.5 ± 1.7	5.2 ± 2.3	0.238
Postoperative	2.6 ± 1.1*	3.3 ± 1.8*	0.241
Final Follow-up	1.2 ± 1.5*	1.5 ± 1.1*	0.256
ODI			
Preoperative	54.7% ± 10.6%	61.6% ± 15.1%	0.091
Postoperative	31.3% ± 10.6%*	35.5% ± 10.5%*	0.206
Final Follow-up	13.9% ± 8.0%*	15.6% ± 9.0%*	0.506
JOA			
Preoperative	16.6 ± 2.8	15.5 ± 3.4	0.166
Postoperative	20.4 ± 3.0*	20.2 ± 2.5*	0.814
Final Follow-up	24.7 ± 5.3*	26.2 ± 5.3*	**0** **.** **043**

Values are presented as mean ± SD or median (interquartile range). Bold indicates *P* < 0.05 between groups.

*indicates *P*<0.05 compared to Preoperative.

### 3.2 Radiographic parameters results

**Figure 4:**
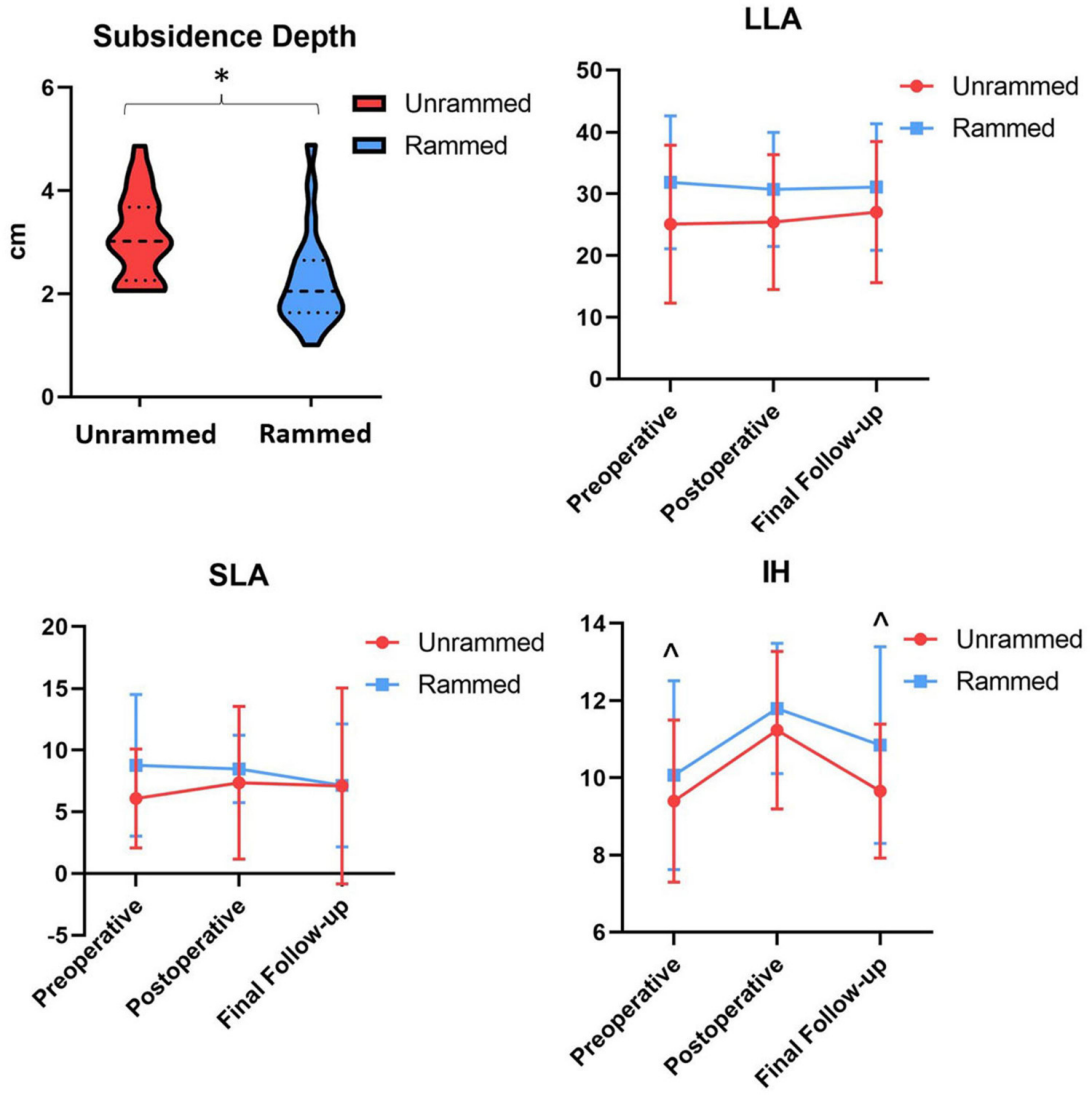
Radiographic parameters charts. LLA, lumbar lordosis angle; SLA, segmental lordosis angle; IH, intervertebral height. ^^^Compared with postoperative, *P* < 0.05.

There was no statistically significant difference in the distance from the posterior fusion edge between the two study groups. (*P* > 0.05). However, the depth of subsidence of the fusion cage was significantly different between the two groups (*P* < 0.05), as was the number of patients who experienced cage subsidence (*P* < 0.05). Postoperative intervertebral height was substantially greater than both preoperative and final follow-up measures (*P* < 0.05) (Figure 4, Table 3). Repeated measures ANOVA indicated no significant differences between the groups in lumbar lordosis angle, segmental lordosis angle, or intervertebral height (*P* > 0.05). Additionally, intervertebral height showed significant differences across different time points (*P* < 0.05). However, no significant interaction effects were observed between the groups over time for lumbar lordosis angle, segmental lordosis angle, or intervertebral distance (*P* > 0.05) (Table 4). No implant-related complications, such as cage or screw displacement, were observed.

**Table 3:** Lumbar measurement-related parameters.

Radiology	Unrammed	Rammed	*P*-value
Subsidence Depth	3.0 (2.2–3.7)	2.1 (1.6–2.7)	**0** **.** **001**
Fusion-edge	7.3 (5.4–8.3)	5.3 (4.0–7.1)	0.055
Subsidence Yes/No	20/1	11/11	**0**.001*
LLA			
Preoperative	25.1 ± 13.1	31.9 ± 10.8	0.070
Postoperative	25.4 ± 11.2	30.7 ± 9.2	0.096
Final Follow-up	27.1 ± 11.7	31.1 ± 10.3	0.240
SLA			
Preoperative	6.1 ± 4.1	8.8 ± 5.7	0.086
Postoperative	7.4 ± 6.3	8.5 ± 2.7	0.435
Final Follow-up	7.1 ± 8.1	7.4 ± 4.9	0.891
IH			
Preoperative	9.4 ± 2.2^^^	10.1 ± 2.4^^^	0.326
Postoperative	11.2 ± 2.1	11.9 ± 1.7	0.271
Final Follow-up	9.7 ± 1.8^^^	10.9 ± 2.5^^^	0.082

LLA, lumbar lordosis angle; SLA, segmental lordosis angle; IH, intervertebral height. Values are presented as mean ± SD or median (interquartile range).

* Significant values for descriptive data were tested with Chi-square analysis. Significant *P*-values are in bold.

^ Compared with postoperative, *P* < 0.05.

**Table 4:** Main effect and interaction.

Outcome variable	Main effect				Interaction	
	Group		Time		Group × Time	
	F-ratio	*P*-value	F-ratio	*P*-value	F-ratio	*P*-value
LLA	2.897	0.097	0.478	0.622	0.897	0.412
SLA	0.924	0.342	0.375	0.673	1.143	0.324
IH	2.526	0.120	16.457	**<0** **.** **001**	0.446	0.641

LLA, lumbar lordosis angle; SLA, segmental lordosis angle; IH, intervertebral height. Significant *P*-values are in bold.

## 4 Discussion

Cage subsidence is one of the complications associated with lumbar fusion surgery and is primarily related to factors such as pressure at the cage-endplate interface, osteoporosis, the quantity of cartilaginous endplate removed during the procedure and the cage's spatial placement (13). Various surgical approaches are available for lumbar fusion, whether performed openly or endoscopically. However, radiographic evidence of cage subsidence remains unavoidable across these approaches. Severe subsidence acts as a catalyst for a deleterious biomechanical cascade: it compromises restored intervertebral height, negating indirect neuroforaminal decompression and potentially precipitating recurrent radiculopathy. Furthermore, asymmetric subsidence drives the loss of segmental lordosis, transferring non-physiological sheer forces onto posterior instrumentation and escalating the risk of hardware failure. To better define subsidence, most studies adopt a threshold of >2 mm as the standard (14). In our study, the subsidence rate in the control group was even higher than that reported in the literature, which may be attributed to the relatively small sample size.

Comparing the JOA scores between two groups, the rammed group showing greater improvement in the final JOA scores. The JOA score is a comprehensive evaluation of lumbar function, reflecting better improvements in subjective symptoms, clinical signs, and daily activities in patients within the rammed group. The lack of differences in short-term VAS and ODI scores between the two groups, along with the absence of surgical complications, indicates that the rammed-earth technique is as safe as the traditional method. In Hiyama et al. study (15), during a one-year follow-up after lumbar fusion, some patients experienced cage subsidence, yet no significant differences were observed in pain, numbness, or other clinical scores. This suggests that subsidence within the first year has minimal impact on symptoms. However, Zhao et al. (16) have followed patients for more than a year post-lumbar fusion surgery, finding that patients with severe cage subsidence (>4 mm) experienced a decline in VAS and ODI scores. Our short observation period and varying sensitivity of the scoring systems might have masked functional differences, leading to the absence of differences in ODI scores.

To prevent cage subsidence, Hung et al. (17) injected bone cement into the vertebral body along the screw trajectory during screw insertion, allowing the bone cement to fill the anterior column of the vertebral body and the endplate in contact with the cage. The injection of bone cement following bone grafting can also provide immediate mechanical support and enhance stability in the short term. However, due to the absence of osseointegration between the cement and host bone, this approach is associated with long-term stress shielding effects (18). With the advancement of 3D printing technology, a novel porous titanium fusion cage has been developed. By optimizing the internal architecture such as stress-optimized lattices and micro-perforated endplates, this design aims to reduce cage stiffness and mitigate stress shielding. Nevertheless, cages produced using this method are relatively expensive, and their mechanical strength and stability under high load conditions may be inferior to those of conventionally solid-designed cages (19). In our results, fusion-edge were similar between groups, while the depth and incidence of cage subsidence differed significantly. In comparison with the aforementioned methods, the rammed-earth technique is relatively simple to perform and associated with lower costs. From a biomechanical perspective, the rammed-earth technique increased the hardness of the implanted bone through hammering and allowed for more bone material to be packed, enhancing the mechanical strength around the cage. This aims to achieve a biomechanical effect similar to structural bone grafting, strengthening the support capacity of the anterior column postoperatively, and reducing compressive forces along the spinal axis. Furthermore, the repeated tamping vibrations continuously rearranged the bone particles, leading to a more uniform distribution of the particles on the endplate, thus more evenly distributing the weight borne along the axis. This reduction in micro-lateral tilting of the cage helps prevent subsequent secondary stress imbalances, reducing the risk of vertebral collapse and minimizing cage subsidence. Previous studies have consistently shown that the bone density of the lower vertebral endplate can predict the degree of cage subsidence (20–22). With varying endplate volumetric bone mineral density (EP-vBMD), a higher elastic modulus of the cage relative to that of the vertebrae can cause cage subsidence. The elastic modulus of materials can be altered by changing porosity (23). The rammed-earth technique likely reduces vertebral subsidence by achieving a better match between the elastic modulus of the fused bone material and the vertebrae. Moreover, the impacted bone graft particles are tightly interlocked with the host bone, thereby increasing the contact area and friction between the graft and surrounding osseous tissue. This enhances the initial stability of the graft and facilitates the interpenetration and ingrowth of osteogenic cells, blood vessels, and other biological components (24). During coronal and sagittal plane movements of the lumbar spine, the increased volume of bone graft particularly in the anterior column suggests that the compacted bone zone created by the rammed-earth technique may more evenly distribute the axial mechanical load. This can reduce stress on the endplates and the cage itself, thereby lowering the risk of cage subsidence caused by stress concentration (25). Previous biomechanical studies have demonstrated that anterior intervertebral support provides superior biomechanical performance during endoscopic lumbar fusion procedures (26). Therefore, in the application of the rammed-earth technique, we placed bone grafts, similar to structural support, in the anterior portion of the intervertebral space to enhance mechanical support. This approach helps to redistribute stress across the interbody cage and adjacent endplates, thereby reducing stress concentration and lowering the risk of cage subsidence.

For lumbar lordosis angle, segmental lordosis angle, and intervertebral height, the main effects of various groups or the interaction effects between group and time showed no statistically significant variations in the repeated measures ANOVA. However, the time effect on intervertebral height was statistically significant. Studies have indicated that changes in lumbar lordosis angle preoperatively, postoperatively, and during follow-up are not significant, while segmental lordosis angle changes substantially after surgical correction and shows little change during subsequent follow-ups, consistent with our findings of no significant time effect on lumbar angle (27). In our analysis, the rammed-earth technique did not significantly affect intervertebral height, which may be related to our measurement method. The rammed-earth technique can result in greater bone graft volume and density between vertebrae, making the intervertebral space boundary more blurred, potentially leading to errors in radiographic measurements in a small sample size. This might explain the lack of significance in intervertebral height results. Pisano et al. (28) found that the height of the implanted segment in the subsidence group was smaller compared to the non-subsidence group following lumbar fusion surgery (28). It can be inferred that, in a larger sample size study, the differences in intervertebral height between the two groups would be more pronounced. In Rickert et al. study (29), 58.2% of patients experienced varying degrees of cage subsidence at one-year follow-up, but it did not affect the progress of fusion, with no correlation found between radiographic results and clinical symptoms. This suggests that subsidence within one year does not impact clinical symptoms. In our study, the JOA scores in the rammed group improved more significantly, possibly related to the height of the implanted segment or the height of intervertebral foramen, warranting further long-term research. The rammed-earth technique involves placing a large amount of compacted bone graft in the anterior part of the intervertebral space. However, compared to traditional surgery without using the rammed-earth technique, this increased bone grafting does not lead to complications of cage posterior displacement into the spinal canal. The absence of a decline in any of the outcome scores indicates the safety and reliability of the rammed-earth technique.

Despite promising outcomes, several methodological limitations warrant acknowledgment. First, the retrospective, single-center design and small cohort size constrain statistical power, increasing the risk of Type II errors for non-significant endpoints like intervertebral height. Second, the 6-month minimum follow-up is a temporal limitation. While acute subsidence appears early, definitive osseous bridging via creeping substitution requires 12–24 months. Thus, delayed subsidence or graft resorption may still occur, confining our conclusions to short- and mid-term stability. Third, retrospective data precluded systematic matching of critical confounding factors, such as inherent bone quality and degeneration severity. Future prospective, adequately powered multicenter trials with extended follow-up (>2 years) and strict bone mineral density stratification are imperative to definitively validate these findings.

Using the rammed-earth technique in PE-P/TLIF procedures can securely reduce the rate and depth of cage subsidence, improving lumbar spine function after a follow-up period of at least six months. However, cage subsidence could not be completely avoided.

## Data Availability

The raw data supporting the conclusions of this article will be made available by the authors, without undue reservation.

## References

[1] Klukowska AM Staartjes VE Vandertop WP Schröder ML. Predictors of five-repetition sit-to-stand test performance in patients with lumbar degenerative disease. Acta Neurochir. (2023) 165:107–15. 10.1007/s00701-022-05441-1 36477416 PMC9840589

[2] Aoki Y Takahashi H Nakajima A Kubota G Watanabe A Nakajima T Prevalence of lumbar spondylolysis and spondylolisthesis in patients with degenerative spinal disease. Sci Rep. (2020) 10:6739. 10.1038/s41598-020-63784-0 32317683 PMC7174286

[3] Liu Z Wang S Li T Chen S Li Y Xie W Clinical efficacy of percutaneous endoscopic posterior lumbar interbody fusion and modified posterior lumbar interbody fusion in the treatment of lumbar degenerative disease. J Orthop Surg Res. (2024) 19:70. 10.1186/s13018-024-04544-y 38225673 PMC10790436

[4] He L-M Chen K-T Chen C-M Chang Q Sun L Zhang Y-N Comparison of percutaneous endoscopic and open posterior lumbar interbody fusion for the treatment of single-segmental lumbar degenerative diseases. BMC Musculoskelet Disord. (2022) 23:329. 10.1186/s12891-022-05287-9 35392878 PMC8988416

[5] Zeng Z-Y Xu Z-W He D-W Zhao X Ma W-H Ni W-F Complications and prevention strategies of oblique lateral interbody fusion technique. Orthop Surg. (2018) 10:98–106. 10.1111/os.12380 29878716 PMC6594526

[6] Kim HW Ryu J-I Bak KH. The safety and efficacy of cadaveric allografts and titanium cage as a fusion substitutes in pyogenic osteomyelitis. J Korean Neurosurg Soc. (2011) 50:348–56. 10.3340/jkns.2011.50.4.348 22200018 PMC3243839

[7] Ge Z He J Zhang P Zhao W Zhu G Zhang J Clinical outcomes and radiologic parameters of endoscopic lumbar interbody fusion using a novel nerve baffle with a Minimum 1-year follow-up. World Neurosurg. (2023) 176:e181–9. 10.1016/j.wneu.2023.05.025 37178917

[8] Li J-R Yan Y Wu X-G He L-M Feng H-Y. Biomechanical evaluation of percutaneous endoscopic posterior lumbar interbody fusion and minimally invasive transforaminal lumbar interbody fusion: a biomechanical analysis. Comput Methods Biomech Biomed Engin. (2024) 27:285–95. 10.1080/10255842.2023.2183348 36847747

[9] Deng Z Zou Q Wang L Wang L Xiu P Feng G Comparison between three-dimensional printed Titanium and PEEK cages for cervical and lumbar interbody fusion: a prospective controlled trial. Orthop Surg. (2023) 15:2889–900. 10.1111/os.13896 37771127 PMC10622287

[10] Calvo-Echenique A Cegoñino J Chueca R Pérez-Del Palomar A. Stand-alone lumbar cage subsidence: a biomechanical sensitivity study of cage design and placement. Comput Methods Programs Biomed. (2018) 162:211–9. 10.1016/j.cmpb.2018.05.022 29903488

[11] You K-H Cho SK Hwang J-Y Cha S-H Kang M-S Park S-M Effect of cage material and size on fusion rate and subsidence following biportal endoscopic transforaminal lumbar interbody fusion. Neurospine. (2024) 21:973–83. 10.14245/ns.2448244.122 39363473 PMC11456953

[12] Kim K-T Park S-W Kim Y-B. Disc height and segmental motion as risk factors for recurrent lumbar disc herniation. Spine. (2009) 34:2674–8. 10.1097/BRS.0b013e3181b4aaac 19910771

[13] Yao Y-C Chou P-H Lin H-H Wang S-T Liu C-L Chang M-C. Risk factors of cage subsidence in patients received minimally invasive transforaminal lumbar interbody fusion. Spine. (2020) 45:E1279–85. 10.1097/BRS.0000000000003557 32472823

[14] Aguirre AO Soliman MAR Kuo CC Kassay A Parmar G Kruk MD Defining cage subsidence in anterior, oblique, and lateral lumbar spine fusion approaches: a systematic review of the literature. Neurosurg Rev. (2024) 47:332. 10.1007/s10143-024-02551-5 39009745

[15] Hiyama A Sakai D Katoh H Nomura S Sato M Watanabe M. Comparative study of cage subsidence in single-level lateral lumbar interbody fusion. J Clin Med. (2022) 11:1374. 10.3390/jcm11051374 35268465 PMC8911078

[16] Zhao L Xie T Wang X Yang Z Pu X Lu Y Clinical and radiological evaluation of cage subsidence following oblique lumbar interbody fusion combined with anterolateral fixation. BMC Musculoskelet Disord. (2022) 23:214. 10.1186/s12891-022-05165-4 35248042 PMC8898418

[17] Hung S-F Tsai T-T Wang S-F Hsieh M-K Kao F-C. Additional cement augmentation reduces cage subsidence and improves clinical outcomes in oblique lumbar interbody fusion combined with anterolateral screw fixation: a retrospective cohort study. Curr Probl Surg. (2024) 61:101441. 10.1016/j.cpsurg.2024.101441 38360009

[18] Cheers GM Weimer LP Neuerburg C Arnholdt J Gilbert F Thorwächter C Advances in implants and bone graft types for lumbar spinal fusion surgery. Biomater Sci. (2024) 12:4875–902. 10.1039/d4bm00848k 39190323

[19] Fogel G Martin N Lynch K Pelletier MH Wills D Wang T Subsidence and fusion performance of a 3D-printed porous interbody cage with stress-optimized body lattice and microporous endplates—a comprehensive mechanical and biological analysis. Spine J. (2022) 22:1028–37. 10.1016/j.spinee.2022.01.003 35017054

[20] Di M Weng Y Wang G Bian H Qi H Wu H Cortical endplate bone density measured by novel phantomless quantitative computed tomography may predict cage subsidence more conveniently and accurately. Orthop Surg. (2023) 15:3126–35. 10.1111/os.13897 37853959 PMC10694013

[21] Pu X Wang X Ran L Xie T Li Z Yang Z Comparison of predictive performance for cage subsidence between CT-based hounsfield units and MRI-based vertebral bone quality score following oblique lumbar interbody fusion. Eur Radiol. (2023) 33:8637–44. 10.1007/s00330-023-09929-x 37462819

[22] Chen Q Ai Y Huang Y Li Q Wang J Ding H MRI-based endplate bone quality score independently predicts cage subsidence following transforaminal lumbar interbody fusion. Spine J. (2023) 23:1652–8. 10.1016/j.spinee.2023.07.002 37442209

[23] Weng Y Di M Wu T Ma X Yang Q Lu WW. Endplate volumetric bone mineral density biomechanically matched interbody cage. Front Bioeng Biotechnol. (2022) 10:1075574. 10.3389/fbioe.2022.1075574 36561040 PMC9763577

[24] Meng B Bunch J Burton D Wang J. Lumbar interbody fusion: recent advances in surgical techniques and bone healing strategies. Eur Spine J. (2021) 30:22–33. 10.1007/s00586-020-06596-0 32949311

[25] Li J Wang W Zuo R Zhou Y. Biomechanical stability before and after graft fusion with unilateral and bilateral pedicle screw fixation: finite element study. World Neurosurg. (2019) 123:e228–34. 10.1016/j.wneu.2018.11.141 30481621

[26] He L Xiang Q Yang Y Tsai T-Y Yu Y Cheng L. The anterior and traverse cage can provide optimal biomechanical performance for both traditional and percutaneous endoscopic transforaminal lumbar interbody fusion. Comput Biol Med. (2021) 131:104291. 10.1016/j.compbiomed.2021.104291 33676337

[27] Choi W-S Kim J-S Hur J-W Seong J-H. Minimally invasive transforaminal lumbar interbody fusion using banana-shaped and straight cages: radiological and clinical results from a prospective randomized clinical trial. Neurosurgery. (2018) 82:289–98. 10.1093/neuros/nyx212 28499016

[28] Pisano AJ Fredericks DR Steelman T Riccio C Helgeson MD Wagner SC. Lumbar disc height and vertebral hounsfield units: association with interbody cage subsidence. Neurosurg Focus. (2020) 49:E9. 10.3171/2020.4.FOCUS20286 32738808

[29] Rickert M Fennema P Wehner D Rahim T Hölper B Eichler M Postoperative cage migration and subsidence following TLIF surgery is not associated with bony fusion. Sci Rep. (2023) 13:12597. 10.1038/s41598-023-38801-7 37537231 PMC10400549

